# Acute visual loss and optic disc edema followed by optic atrophy in two cases with deeply buried optic disc drusen: a mimicker of atypical optic neuritis

**DOI:** 10.1186/s12886-018-0949-1

**Published:** 2018-10-26

**Authors:** Mário Luiz R. Monteiro, Kenzo Hokazono, Leonardo P. Cunha, Laurentino Biccas Neto

**Affiliations:** 1 Division of Ophthalmology and Laboratory of Investigation in Ophthalmology (LIM 33), University of São Paulo Medical School, São Paulo, Brazil; 2 Federal University of Paraná, Curitiba, Paraná Brazil; 3 Federal University of Juiz de Fora, Minas Gerais, Brazil; 4 Ocular Oftalmologia, Vitória, Brazil

**Keywords:** Optic disc drusen, Optical coherence tomography, Optic neuritis, Neuromyelitis optica, Ischemic optic neuropathy

## Abstract

**Background:**

Sudden visual loss and optic disc edema caused by optic neuritis (ON) is usually followed by significant visual recovery. However, little or no recovery occurs when the loss is caused by atypical ON, especially in patients with neuromyelitis optica (NMO). Optic disc drusen (ODD) is a cause of pseudo optic disc edema and may be a predisposing factor for non-arteritic anterior ischemic optic neuropathy (NAION), thereby mimicking atypical ON. In such cases, if globular concretions are seen protruding from the disc substance, ODD may be suspected. The purpose of this paper is to describe two patients with acute visual loss followed by optic disc atrophy initially labeled as atypical ON. Though not suspected on clinical examination, optical coherence tomography (OCT) revealed deeply buried ODD as a predisposing factor for NAION.

**Case presentations:**

Case 1: A 48-year-old woman had bilateral sequential visual loss associated with optic disc edema. Despite treatment, vision did not improve and severe disc pallor ensued. Atypical ON was suspected. Eventually, she was started on immunosuppressant therapy based on a tentative diagnosis of NMO-spectrum disorder. On examination 5 years later, only severe optic disc pallor was observed, but OCT radial B-scans showed ovoid hyporeflective areas in the retrolaminar region of both eyes, compatible with ODD; this led to a diagnosis of NAION and deeply buried ODD. Case 2. A 35-year-old woman with suspicion of ON in the left eye and a history of previous atypical ON in the right eye was referred for neuro-ophthalmic examination which revealed diffuse optic disc pallor and a dense arcuate visual field defect in the right eye. OCT B-scans passing through the disc showed large ovoid areas of reduced reflectivity in the retrolaminar region of the optic disc in the right eye. These findings helped confirm the diagnosis of NAION in one eye, with deeply buried ODD as predisposing factor.

**Conclusions:**

Deeply buried ODD may be associated with NAION causing irreversible visual loss and optic disc pallor, a condition easily mistaken for atypical ON. Awareness of such occurrence is important to avoid unnecessary testing and minimize the risk of mismanagement.

## Background

Acute visual loss and optic disc edema in non-senile patients is generally due to optic neuritis (ON), an inflammatory/demyelinating disease which often resolves with visual improvement in a matter of weeks [1]. Atypical ON, on the other hand, is associated with severe forms of ON and may lead to permanent visual loss, especially in patients with neuromyelitis optica (NMO) [1, 2].

Optic disc drusen (ODD) are laminated and usually calcified acellular globular concretions protruding from the optic disc or hidden within the disc substance. ODD near the surface of the disc are clearly visible on fundus examination but, when located below the retinal nerve fiber layer (RNFL), may alter the contour of the optic disc and mimic true optic disc edema [3]. Though usually a benign condition, ODD can be associated with acute visual loss due retinal vascular complications or non-arteritic anterior ischemic optic neuropathy (NAION) [4]. In such cases, diagnostic confusion with atypical ON may occur, but the presence of ODD generally can be detected or suspected on fundus examination (especially after resolution of optic disc edema) and confirmed with appropriate ancillary testing, including B-scan ultrasonography, autofluorescence imaging, computerized tomography [5] and optical coherence tomography (OCT) [3].

Recently, however, high-resolution OCT studies have shown that ODD deeply buried in the optic disc structure easily escapes clinical detection, even by experienced examiners [3]. We examined two patients that presented with optic disc edema and acute visual loss unresponsive to treatment and followed by severe optic atrophy. ODD was not suspected on fundus examination. Due to the sequential involvement of the second eye in one case and the young age of the other, both patients were tentatively diagnosed with atypical ON, presumably from NMO-spectrum disease. However, OCT imaging of the retrolaminar space of the disc revealed deeply buried ODD as a predisposing factor for NAION. Awareness of this mimicker is important to avoid unnecessary testing and potentially harmful treatment.

## Case presentations

### Case 1

A previously healthy 48-year-old woman developed sudden and painless visual loss and optic disc edema in the right eye (OD). A tentative diagnosis of ON was made and high-dose intravenous methylprednisolone was administered for 5 days, followed by oral prednisone therapy, but to no avail. Three weeks later a similar event occurred in the left eye (OS). Oral corticosteroid treatment was maintained for the following 4 months with only slight improvement in vision. The disc edema resolved and was followed by severe optic atrophy. Neurologic examination, brain and orbits computerized tomography and magnetic resonance imaging (MRI) scans, cerebrospinal fluid (CSF) analysis and extensive laboratory investigation including anti-aquaporin 4 antibody assay were unrevealing, except for a weakly positive antinuclear antibody test (1/160, granular pattern). Because of sequential involvement and the lack of significant improvement the patient was considered to have atypical ON, presumably from NMO-spectrum disease. Oral azathioprine 150 mg/day was introduced and kept for the following years. Her visual function remained stable, and significant peripapillary RNFL loss developed on sequential spectral-domain (SD) OCT examinations, directed at quantifying peripapillary RNFL. However, high-resolution OCT scanning passing through the optic nerve were not obtained.

**Fig. 1:**
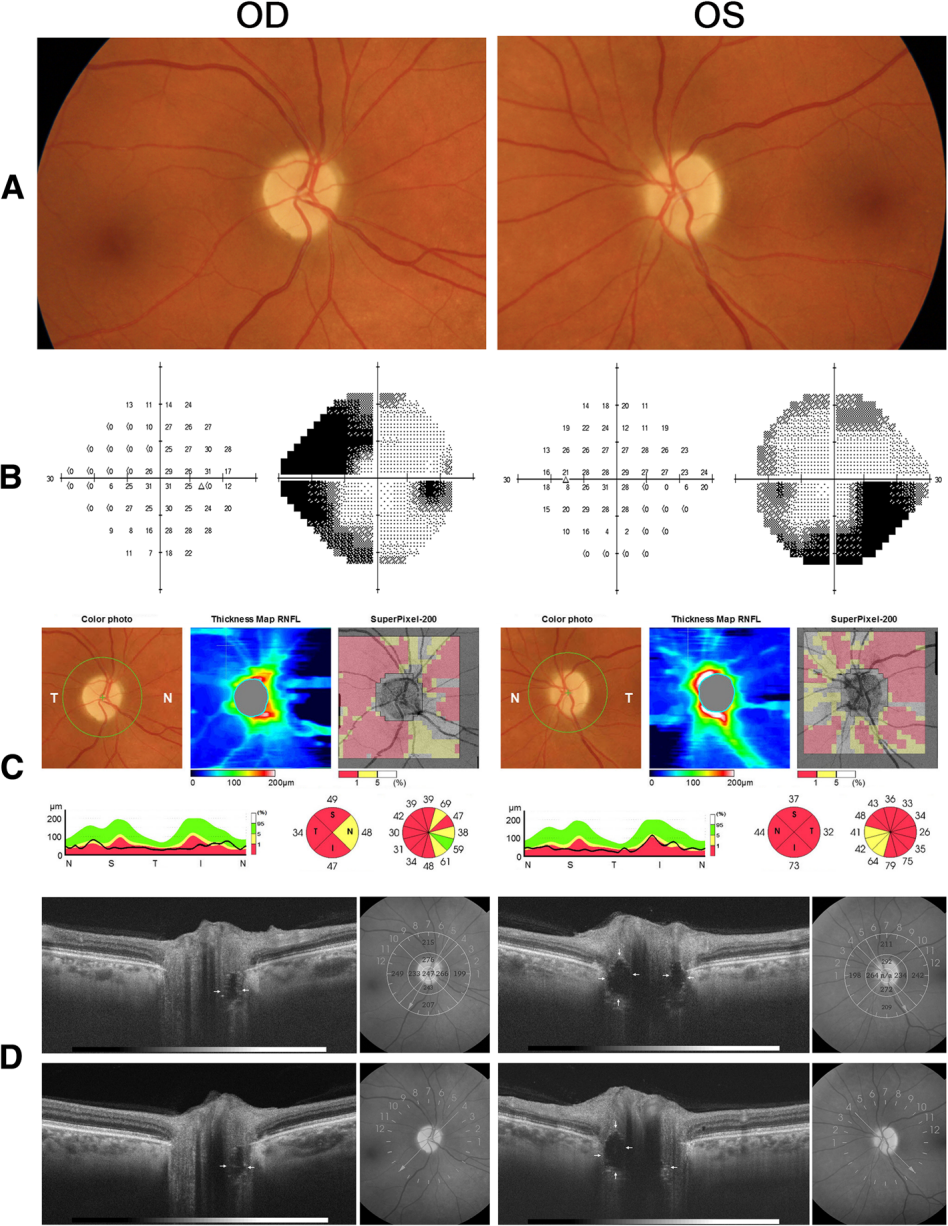
**a** Fundus photograph showing diffuse optic disc pallor in both eyes (OU); **b** visual field on 24–2 automated perimetry (Humphrey 24–2, SITA Standard test) showing severe visual loss OU; **c** swept-source optical coherence tomography (DRI OCT Triton Plus®; Topcon corporation, Tokyo, Japan) showing severely reduced peripapillary retinal nerve fiber layer OU, and **d** radial OCT B-scans passing through the optic disc showing deeply buried ovoid structures (arrows) surrounded by hyper-reflective bands OU

Five years later, the patient was seen for the first time by us for a second opinion regarding her condition and her current treatment. Her vision was stable and she had no new complaints. Upon examination, visual acuity (VA) was 20/20–2 in OD and 20/25 in OS. A mild relative afferent pupillary defect was present in OS. Extraocular motility, slit lamp findings and intraocular pressure measurements were normal. The fundus examination showed diffuse disc pallor in both eyes (OU). The visual field (VF) test revealed marked sensitivity loss in the upper and lower nasal quadrants, enlarged blind spot in OD, and marked lower nasal and temporal defect associated with diffuse VF depression in OS. Swept-source (SS) OCT showed severely reduced peripapillary RNFL thickness in OU. OCT radial B-scans passing through the disc showed ovoid areas of reduced reflectivity with some hyper-reflective areas in the retrolaminar region of the disc in both eyes (Fig. 1). The findings were consistent with a diagnosis of ODD complicated by NAION. Azathioprine treatment was discontinued and she was advised to continue periodic ophthalmic examination.

### Case 2

A 35-year-old woman presented with a complaint of a small dark spot laterally in OS. Taking into account her history of acute visual loss in OD 10 years earlier, left optic disc edema from ON was suspected by her ophthalmologist and the patient was referred to us for neuro-ophthalmic evaluation. Ten years previously, she had acute VF loss and optic disc swelling in OD which regressed completely after treatment with intravenous high-dose methylprednisolone for 5 days. However, since her visual loss remained unchanged she was tentatively diagnosed with atypical ON by her previous physicians. After extensive laboratory testing including anti-aquaporin-4 antibody, MRI and CSF examination were normal, close neurological follow-up was prescribed justified by the suspicion of seronegative NMO-spectrum disease.

**Fig. 2:**
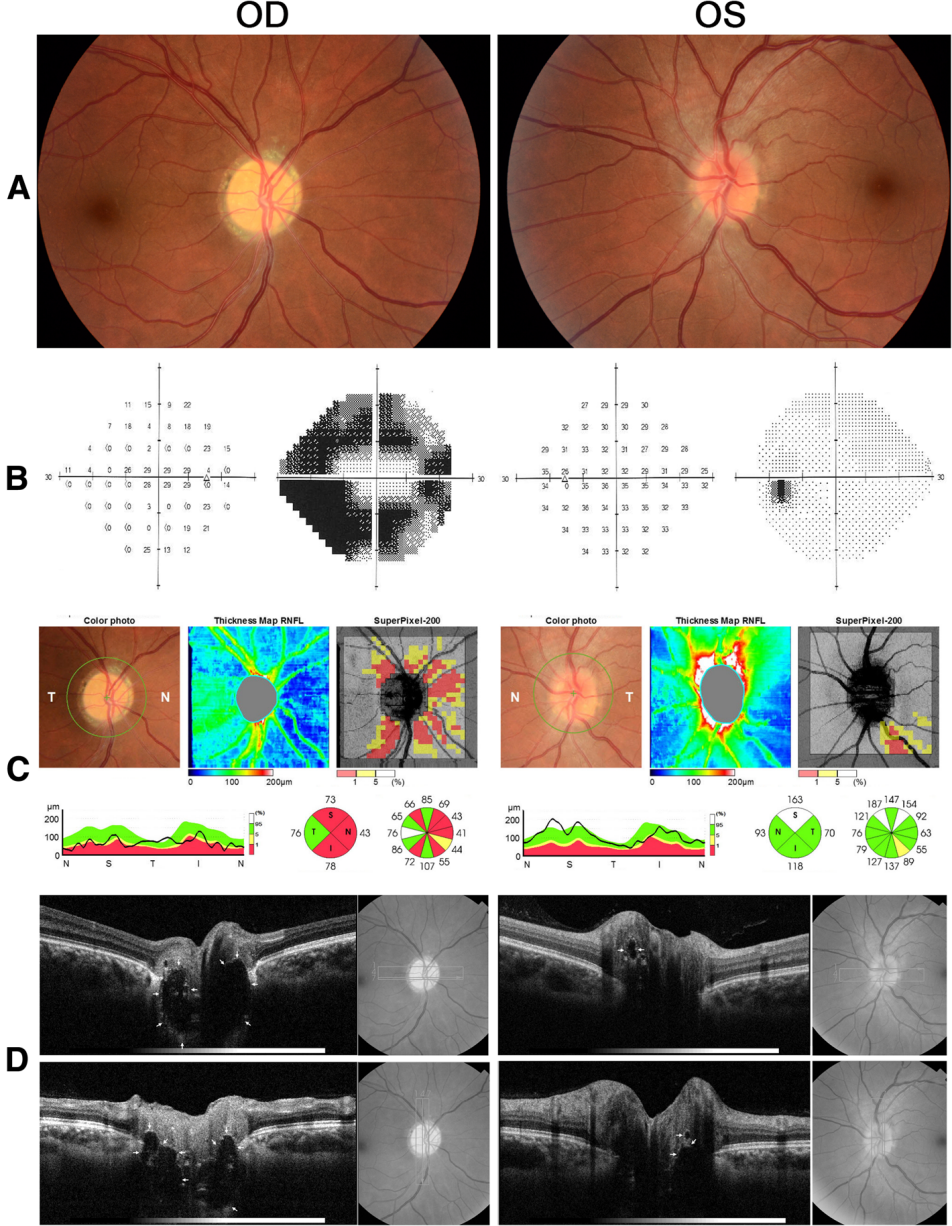
**a** Fundus photograph showing diffuse optic disc pallor in the right eye (OD) and mildly blurred disc in the left eye (OS); **b** Visual field (Humphrey 24–2 SITA Standard test) showing severe defect OD and normal field OS; **c** spectral-domain optical coherence tomography (OCT-2000®, Topcon corporation, Tokyo, Japan) showing severely reduced peripapillary retinal nerve fiber layer OD, and **d** enhanced-depth OCT horizontal and vertical B-scans passing through the optic disc showing ovoid structures (arrows) surrounded by hyper-reflective bands deeply buried in OD and superficially located in OS

Upon ophthalmic examination, VA was 20/20 in OU and the pupils reacted to light and near stimuli with a relative afferent pupillary defect in OD. Extraocular motility, slit lamp examination and intraocular pressure measurements were normal. The fundus examination showed diffuse optic disc pallor with a peripapillary depigmented halo around the disc in OD and mildly blurred disc margins in OS. A small round vitreous opacity presumably due to vitreous detachment was found in OS. The VF examination disclosed an arcuate dense pericentral defect in OD and was within normal range in OS. Spectral-domain (SD) OCT showed severely reduced peripapillary RNFL thickness in OD and mildly increased RNFL thickness in the superior quadrant of OS. Enhanced-depth imaging (EDI) horizontal and vertical OCT B-scans passing through the disc showed large ovoid areas of reduced reflectivity with some hyper-reflective regions in the retrolaminar region of the optic disc in OD (Fig. 2). The OCT scans also revealed a small prelaminar area of reduced reflectivity and some deeper areas in OS, suggesting ODD in both eyes, complicated by NAION in OD. Mildly increased RNFL thickness in OS was attributed to ODD and her recent complaint of a small dark spot in her vision was due to vitreous opacity. No evidence of NAION in OS was found and the appearance of her disc remained stable on follow-up examinations.

## Discussion

Subtle and insidious visual loss is relatively common manifestation of ODD, usually characterized by subclinical VF defect associated with peripapillary RNFL loss [6]. ODD are associated with VF defect, with a reported incidence ranging from 24 to 87% of affected adults [3, 7]. In most such cases, the visual deficit takes the form of slowly progressive VF defects that may resemble glaucomatous visual loss and is often unnoticed by the patient [4]. On the other hand, sudden or severe visual loss in eyes with ODD is unusual, but may occur in association with complications such as NAION [4, 8], retinal hemorrhage and subretinal neovascular membrane [9].

NAION associated with ODD usually occurs in middle aged or senile patients, characterized by altitudinal VF loss, optic disc edema and occasional peripapillary hemorrhages, without significant visual improvement. In such cases, ODD is considered a predisposing factor for NAION and its presence is generally detected either in the early stages of visual loss or after resolution of optic disc edema. The observation of characteristic globular concretions protruding from the optic disc or within the prelaminar region of the optic nerve head is usually enough to establish the correct diagnosis. While further testing may be necessary to confirm it [3, 5], ODD can often be suspected on clinical grounds, especially when optic disc edema subsides.

The pathogenesis of NAION in eyes with ODD in not completely understood but is thought to involve a decrease in the perfusion pressure at the optic nerve head that leads to infarction [10]. Both NAION and ODD usually occurs in eyes with absent or small cup-disc ratio and crowded optic appearance [4, 11–13]. While the occurrence of both conditions might be coincidental it is most likely that ODD contributes for the development of NAION by promoting further crowding the disk contributing to secondary vascular compromise [4]. Purvin et al. [4] reviewed the clinical findings in 20 patients (24 affected eyes) that experienced NAION associated with ODD and found many similarities with those of NAION in eyes unassociated with drusen. However, patients with ODD were younger, fared a more favorable visual outcome and were more likely to report preceding episodes of transient visual obscuration. Some of these characteristics are present in our cases; case 2 had visual loss at age 25 and VA was 20/25 or better in the three affected eyes. As for the diagnosis of ODD, in 7 eyes it was established on fundus examination and in the others it was established on ultrasonography or computed tomography after the clinical suspicion because of disc appearance had a bumpy surface, scalloped margins or deep peripapillary hemorrhage [4].

Our cases are interesting because ODD was too deep to be suspected on clinical examination, even after optic disc edema resolved. Although NAION was included in the differential diagnosis of ON, the sequential involvement and a weakly positive antinuclear antibody titer in one patient and the young age of the other patient were strongly suggestive of atypical ON. On the other hand, none had ocular pain, significant VA loss or progressive visual loss that are commonly associated with ON [1, 2]. However, because atypical ON was considered the most likely explanation, an extensive investigation for NMO and multiple sclerosis was carried out and, in one case, prolonged immunosuppressive treatment was administered. Once deeply buried ODD was detected, the diagnosis of NAION with ODD as a predisposing factor could be entertained. Although both patients had been submitted to OCT for quantification of RNFL loss, the correct diagnosis could only be established after radial swept-source or enhanced-depth SD-OCT scans had been performed. In case #2, deeply buried ODD in the unaffected eye (OS) might have been suspected on further observation, but the affected eye and both eyes of case #1 only displayed severe optic disc pallor, with no suggestion of ODD. However, since atypical ON was not supported by the finding of relatively preserved VA and the pattern of VF defect in both patients, we persisted in the search for an alternative diagnosis.

OCT is currently considered by many authors as the best method for detecting ODD [14–16], superior to that of ultrasonography which previously was considered the best-performing diagnostic modality of the detection of buried ODD [5]. Because it is a non-invasive imaging technique and allows for the detection of not only calcified but also non calcified ODD, OCT has largely replaced the other imaging techniques in its detection. When SD-OCT is used for investigation of ODD, there is a current consensus that EDI high-resolution, cross-sectional images are the ideal protocol for diagnosis [14]. Another important technology for detecting deep ODD is OCT with the SS technology, which uses a light source with a wavelength of 1,050 nm, with high-penetration that allows high-definition images of the deep optic disc structures [3, 16]. While no comparative study between the two technologies both appear to be equally sensitive for detecting ODD, provided that multiple high-resolution cross-sectional images are obtained. There is a consensus that ODD is detected on OCT as hyporeflective structures with hypereflective margin [3, 14], as demonstrated in our cases. Many authors consider that isolated hypereflective bands on OCT can also considered to be indicative of early ODD [15, 16]. However, it is important to point out that OCT with low-resolution cross-sectional images of the disc without EDI strategy (for SD-OCT) or SS technology not infrequently fail to detect deeply located. This can be exemplified by our case 1 that had already been submitted to sequential evaluations with SD-OCT previously but without strategies for specifically searching for deeply-located ODD. This caveat is important to avoid diagnostic confusion.

## Conclusion

In conclusion, our cases show that, while clinically difficult to detect, deeply buried ODD may play an important part in the etiology of acute irreversible visual loss and optic disc edema. Ophthalmologists should be aware of this mimicker of atypical ON in order to avoid unnecessary testing and minimize the risk of mismanagement. The current cases also emphasize the utility of modern OCT technology in correctly detecting ODD, even when it is deeply buried in the optic disc.

## Abbreviations

CSF: Cerebrospinal fluid
MRI: Magnetic resonance imaging
NAION: Non-arteritic ischemic optic neuropathy
NMO: Neuromyelitis optica
OCT: Optic coherence tomography
OD: Right eye
ODD: Optic disc drusen
ON: Optic neuritis
OS: Left eye
OU: Both eyes
RNFL: Retinal nerve fiber layer
VA: Visual acuity
VF: Visual field
